# T61. ANTISACCADE AND MEMORY GUIDED SACCADE PERFORMANCE ACROSS THE SCHIZOPHRENIA CONTINUUM

**DOI:** 10.1093/schbul/sby016.337

**Published:** 2018-04-01

**Authors:** Elizabeth Thomas, Susan Rossell, Eric Tan, Sean Carruthers, Philip Sumner, Kiymet Bozaoglu, Caroline Gurvich

**Affiliations:** 1 Monash Alfred Psychiatry Research Centre; 2 Monash Alfred Psychiatry Research Centre, Swinburne University, St. Vincent’s Hospital; 3 Swinburne University, Monash Alfred Psychiatry Research Centre, The Alfred Hospital; 4 University of Melbourne; 5 Monash Alfred Psychiatry Research Centre, The Alfred Hospital, Monash University

## Abstract

**Background:**

Saccadic (ocular motor) deficits are one of the most replicated findings in schizophrenia. However, less research has been conducted investigating the broader schizophrenia continuum. Recent research suggests that the personality characteristics and symptoms observed in schizophrenia lie on a continuum with subclinical symptoms, known as schizotypy, observed in the non-clinical population. Schizotypy is considered a suitable model for investigating schizophrenia as it mirrors the symptoms, albeit in a more subtle manner. As saccadic deficits are a cognitive hallmark of schizophrenia, it is believed that saccadic deficit may be associated with higher schizotypy. The aim of the current study was to 1) replicate previous findings of impairments in antisaccade and memory-guided saccade performance in schizophrenia and 2) investigate the relationship between antisaccade and memory-guided saccade performance and schizotypy.

**Methods:**

105 adults (35 patients with schizophrenia/schizoaffective disorder and 70 healthy controls) completed the antisaccade and memory-guided saccade tasks, which engage spatial working memory and inhibition processes. The variables analysed for both saccade paradigms were error rate, latency (ie. reaction time) and gain (ie. spatial accuracy). Schizotypy was assessed using the Oxford-Liverpool Inventory of Feelings and Experiences (O-LIFE), a 104 item questionnaire which measures the three main schizotypy factors: Unusual Experiences, Introvertive Anhedonia and Cognitive Disorganisation. A total O-LIFE score was also calculated from these three schizotypy factors as a representation of global schizotypy. Controls were then divided into low (n = 35) and high schizotypy groups (n = 35). A MANOVA was conducted to observe differences in eye movement variables between low schizotypy individuals, high schizotypy individuals and patients. Correlations were also conducted to further investigate these relationships.

**Results:**

Antisaccade error rate, (p < 0.001), antisaccade latency (p = 0.007), memory-guided saccade error rate (p = 0.009) and latency (p < 0.001) were significantly different between patients and controls. When comparing low schizotypy, high schizotypy and patient groups, the MANOVA revealed significant differences for antisaccade and memory-guided saccade latency and a non-significant trend for antisaccade gain. However, post-hoc analyses revealed that there was only a significant difference between low schizotypy and patient groups (p < 0.001), but not between low schizotypy and high schizotypy nor between high schizotypy and patient. Looking across the schizophrenia continuum, there were significant correlations between the total O-LIFE score and antisaccade gain (p = 0.033), memory-guided saccade latency (p = 0.014) and memory-guided saccade gain (p = 0.011). A non-significant trend was also observed between the total O-LIFE score and antisaccade latency (p = 0.088).

**Discussion:**

This study replicated previous findings of impaired saccade performance in schizophrenia. In addition, it also replicated findings of impaired antisaccade performance in higher schizotypy and is the first study to investigate and demonstrate the relationship between higher schizotypy and impaired memory-guided saccade performance. Overall, these findings supporting the use of schizotypy as a model for schizophrenia and also support the theory of schizotypy and a broader schizophrenia continuum.

